# TNF‐α/HMGB1 inflammation signalling pathway regulates pyroptosis during liver failure and acute kidney injury

**DOI:** 10.1111/cpr.12829

**Published:** 2020-05-17

**Authors:** Yao Wang, Haiyue Zhang, Qian Chen, Fangzhou Jiao, Chunxia Shi, Maohua Pei, Jian Lv, Hong Zhang, Luwen Wang, Zuojiong Gong

**Affiliations:** ^1^ Department of Infectious Diseases Renmin Hospital of Wuhan University Wuhan China; ^2^ Department of Pharmacy Renmin Hospital of Wuhan University Wuhan China

**Keywords:** acute kidney injury, HMGB1, liver failure, macrophages, pyroptosis, TNF‐α

## Abstract

**Objective:**

Acute kidney injury (AKI) is a common complication of acute liver failure (ALF). Pyroptosis is a necrosis type related to inflammation. This study aimed to investigate the role of TNF‐α/HMGB1 pathway in pyroptosis during ALF and AKI.

**Methods:**

An ALF and AKI mouse model was generated using LPS/D‐Gal, and a TNF‐α inhibitor, CC‐5013, was used to treat the mice. THP‐1 cells were induced to differentiate into M1 macrophages, then challenged with either CC‐5013 or an HMGB1 inhibitor, glycyrrhizin. pLVX‐mCMVZsGreen‐PGK‐Puros plasmids containing TNF‐α wild‐type (WT), mutation A94T of TNF‐α and mutation P84L of TNF‐α were transfected into M1 macrophages.

**Results:**

Treatment with CC‐5013 decreased the activation of TNF‐α/HMGB1 pathway and pyroptosis in the treated mice and cells compared with the control mice and cells. CC‐5013 also ameliorated liver and kidney pathological changes and improved liver and renal functions in treated mice, and the number of M1 macrophages in the liver and kidney tissues also decreased. The activation of TNF‐α/HMGB1 pathway and pyroptosis increased in the M1 macrophage group compared with the normal group. Similarly, the activation of TNF‐α/HMGB1 pathway and pyroptosis in the LPS + WT group also increased. By contrast, the activation of the TNF‐α/HMGB1 pathway and pyroptosis decreased in the LPS + A94T and LPS + P84L groups. Moreover, glycyrrhizin inhibited pyroptosis.

**Conclusion:**

The TNF‐α/HMGB1 inflammation signalling pathway plays an important role in pyroptosis during ALF and AKI.

## 1 INTRODUCTION

Acute liver failure (ALF) is a clinically common severe liver disease syndrome caused by various factors and could lead to serious disorders and decompensation of liver synthesis, detoxification, excretion and biotransformation. The main clinical manifestations of ALF are coagulopathy, jaundice, liver sexual encephalopathy and ascites. Supportive care and liver transplantation are the main treatment strategies for ALF^1^; however, the mortality rate is still high. The kidneys are one of the most vulnerable extrahepatic organs in patients with liver failure. Acute kidney injury (AKI) is a group of clinical syndromes characterized by renal tubular injury, inflammation and vascular dysfunction.^2^ AKI usually results in an increase in mortality in patients with liver failure. Therefore, prevention and treatment of AKI is very important in controlling the progression of liver failure.

Infection is one of the main causes of AKI in patients with liver failure. Once the kidney injury occurs after infection, the mortality rate increases dramatically.^3^ The occurrence of AKI is significantly associated with severity of the systemic inflammatory reaction syndrome (SIRS).^4^, ^5^, ^6^ Due to weakened immune system in ALF patients, the function of the intestinal mucosal barrier is reduced. Overgrowth of intestinal bacteria releases intestinal endotoxins, which not only induces a large number of necrotic hepatocytes through the hepato‐intestinal circulation, but also activates liver macrophages to release pro‐inflammatory factors.^7^ The decreased hepatocyte clearance and activation of macrophages lead to increased levels of endotoxins and inflammatory factors in circulation, which may further cause kidney damage.

The term “pyroptosis,” which refers to a cysteinyl aspartate‐specific proteinase 1 (caspase‐1)‐dependent cell death pattern, was first proposed by Cookson et al in 2001.^8^ During the process of pyroptosis, nucleotide‐binding oligomerization domain‐like receptor protein 3 (NLRP3) combines with cysteine protease pro‐caspase‐1 to form inflammasomes. Inflammasomes can process pro‐caspase‐1 into mature caspase 1.^9^ Furthermore, cleavage and activation of the pore‐forming effector protein gasdermin D (GSDMD) by activated caspase is an important process in the induction of pyroptosis, during which pro‐inflammatory cytokines, such as IL‐1β and IL‐18, are released from the damaged cells. Immune cells are activated by the pro‐inflammatory cytokines, which in turn, induce pyroptosis, leading to a vicious cycle.^10^ Pyroptosis occurs mainly in Kupffer cells or kidney macrophages.^11^, ^12^, ^13^ As important immune cells, macrophages play a critical role in the process of phagocytosis and digestion of pathogenic bacteria. Studies have shown that the large number of inflammatory mediators released by M1 macrophages is one of the important causes of organ damage in sepsis.^14^ The TNF‐α gene is located on the short‐arm major histocompatibility complex (MHC) Ⅲ region of human chromosome 6. Several studies have demonstrated that the TNF‐α promoter region contains numerous single nucleotide polymorphisms (SNPs), including 238G/A, 244G/A, 308G/A, 376G/A, 575G/A, 857C/T, 863C/A and 1031T/C.^15^, ^16^, ^17^ Such SNPs can affect the transcriptional activity and expression of the TNF‐α gene.^18^, ^19^ Moreover, SNPs of TNF‐α are related to multiple inflammatory and autoimmune diseases, including rheumatoid arthritis,^20^ hepatitis B^21^ and lupus nephritis.^22^

As a late inflammatory mediator, high mobility group box 1 (HMGB1) responds to the early inflammatory mediator (TNF‐α), thereby maintaining and prolonging inflammatory responses. Our previous study has suggested that HMGB1 is involved in the inflammatory response during ALF.^23^ Meanwhile, it was reported that the A94T/P84L SNPs of TNF‐α could affect the activation of hepatic stellate cells.^23^ However, the mechanisms of action of TNF‐α/HMGB1 inflammation signalling pathway and A94T/P84L SNPs of TNF‐α in pyroptosis during ALF and AKI remain unknown till date. Lenalidomide (CC‐5013), a small molecule, is an inhibitor of TNF‐α,^24^ and its main function is to inhibit the secretion of TNF‐α production.^24^, ^25^ Thus, this study aimed to investigate the role of TNF‐α/HMGB1 inflammation signalling pathway in pyroptosis during ALF and AKI by using CC‐5013 and SNPs of TNF‐α.

## 2 MATERIALS AND METHODS

### 2.1 Reagents

Foetal bovine serum (FBS) and RPMI1640 were purchased from HyClone. The pyroptosis/caspase‐1 kit was obtained from Immunochemistry. Lipopolysaccharides (LPS), phorbol 12‐myristate 13‐acetate (PMA), D‐galactosamine (D‐Gal) and TNF‐α were purchased from Sigma. The TNF‐α inhibitor, CC‐5013, was purchased from Selleckchem and the HMGB1 inhibitor, glycyrrhizin, from MedChemExpress (MCE). HMGB1, TNF‐α, IL‐1β and IL‐18 enzyme‐linked immunosorbent assay (ELISA) kits were procured from Elabscience. RNAiso Plus, PrimeScript RT reagent and SYBR Premix Ex Taq kits were purchased from TAKARA. Primary antibodies to inducible nitric oxide synthase (iNOS), CD68, mannose receptor (MR), arginase‐1 (Arg‐1), NLRP3, GSDMD, IL‐1β, IL‐18 and GAPDH were purchased from ProteinTech. Rabbit anti‐human primary antibody pro‐caspase‐1 + p10 + p12 was procured from Abcam and IRDye800CW secondary antibody from LI‐COR Biosciences.

### 2.2 Cell culture, plasmid constructs and intervention

Human monocyte cell line, THP‐1, was purchased from the Cell Collection Center of Wuhan University (Wuhan, China). 1640 medium containing 10% serum was used for in vitro culture of THP‐1 cells, which were cultured in an incubator with 5% CO_2_ at 37°C. After cell confluency reached 70%, the cells were stimulated with 50 ng/mL PMA for 48 hours. When THP‐1 cells attach to the walls of the cell culture flask and grow pseudopods, they are said to be differentiated into macrophages. As reported in our previous study,^23^ pIRES2‐ZsGreen1 plasmids encoding TNF‐α wild‐type, mutation A94T of TNF‐α and mutation P84L of TNF‐α were obtained from Wuhan Biofavor Biotechnology Co. Ltd. The TNF‐α, TNF‐α/A94T and TNF‐α/P84L inserts were subcloned into pLVX‐mCMVZsGreen‐PGK‐Puro. Next, M1 macrophages were transfected with these plasmids using the Lenti‐Pac^™^ system. A normal control (NC) plasmid was also constructed. The macrophages were divided into normal, LPS, LPS + WT, LPS + P84L, LPS + A94T and LPS + NC groups. Except for the normal group, the other groups were stimulated with 1 μg/mL LPS. THP‐1 cells stimulated by PMA were treated with TNF‐α for 24 hours. Glycyrrhizin was then added as an intervening factor for 24 hours before the cells were harvested.

### 2.3 Mouse models and drug administration

Thirty C57/BL6 male mice weighing 20‐25 g (8‐10 weeks old) were obtained from the Hubei Provincial Center for Disease Control and Prevention (Wuhan, China). Mice were adapted to the new experimental environment for 3 days and received humanistic care according to the Guide for the Care and Use of Laboratory Animals of Institutional Animal Care and Use Committee of Renmin Hospital of Wuhan University. The mice were divided into normal, ALF and AKI model, and CC‐5013 groups. The ALF and AKI mouse model was intraperitoneally injected with LPS (100 μg/mL) and D‐Gal (400 mg/mL) for 24 hours. Besides, CC‐5013 (3 mg/kg) was administered to mice for 2 hours to generate mice in the CC‐5013 group. The same amount of saline was administrated to mice in the normal and model groups, at that same time.

### 2.4 Histological and immunofluorescence detection

Liver and kidney specimens were fixed in 4% polyformaldehyde for 24 hours and then processed for sectioning and staining. Haematoxylin and eosin (H&E) staining procedure was performed as previously described,^26^ and pathological changes in the liver and kidney were evaluated under a light microscope. Additionally, the sections were incubated with iNOS and CD68 antibodies overnight, followed by incubation with secondary antibodies for 1 hour. Furthermore, the slides were counterstained with a nuclear dye (DAPI). The expression and location of iNOS and CD68 were observed under a fluorescence microscope.

### 2.5 Biochemical tests and detection of cytokines

Serum alanine aminotransferase (ALT), aspartate aminotransferase (AST), total bilirubin (TBil), blood urea nitrogen (BUN) and creatinine (Cr) levels were estimated using a fully automated Aeroset chemistry analyser provided by Abbott Co. Ltd. The levels of TNF‐α, HMGB1, IL‐1β and IL‐18 in serum and cell supernatants were detected using ELISA kits, according to the manufacturer's instructions.

### 2.6 Quantitative real‐time PCR (qRT‐PCR) to detect TNF‐α mRNA levels

Total RNA was isolated from the cells using RNAiso Plus. Next, the RNA samples were reversed transcribed into cDNA using a Prime‐Script RT reagent kit. qRT‐PCR was performed using a SYBR Premix Ex Taq kit on a StepOnePlus device (Applied Biosystems). The data were analysed by the 2^−ΔΔCT^ method. The forward and reverse primer sequences of TNF‐α were 5′‐TCCTTCCTGATCGTGGCA‐3′ and 5′‐TGAAGAGGACCTGGGAGTAGAT‐3′, respectively, and those of housekeeping gene GAPDH were 5′‐ATGACATCAAGAAGGTGGTG‐3′ and 5′‐CATACCAGGAAATGAGCTTG‐3′, respectively.

### 2.7 Protein levels of NLRP3, caspase‐1, GSDMD, IL‐1β and IL‐18 were detected by Western blotting

Total proteins from cells or tissues were extracted, measured and separated by SDS‐PAGE, and then transferred to PVDF membranes. The membranes were blocked with skim milk and were later incubated overnight at 4°C with rabbit anti‐human primary antibodies against pro‐caspase‐1 + p10 + p12 (1:800), GSDMD (1:1000), IL‐1β (1:1000), IL‐18 (1:1000), NLRP3 (1:1000) and GAPDH (1:1000). After washing with the Tris‐buffered saline with Tween 20 (TBST), the membranes were incubated with goat anti‐rabbit IRDye800CW secondary antibody at room temperature in the dark. The membranes were analysed using the Odyssey Infrared Imaging System (LI‐COR Biosciences).

### 2.8 The rate of pyroptosis was detected by flow cytometry

The cells in each group were washed and collected. According to the instructions provided in the pyroptosis/caspase‐1 pyroptosis kit, the cells were resuspended in 500 μL binding buffer. Next, 5 μL FAM‐YVAD‐FMK‐FLICA and 5 μL PI were mixed in the cell resuspension for 15 minutes at room temperature in the dark. Flow cytometry (BD) was used to detect the pyroptosis rate.

### 2.9 Statistical methods

Data are expressed as mean ± standard deviation. The data were analysed by Student's *t* test and one‐way analysis of variance (ANOVA) employing the SPSS 13.0 software. A *P* value < .05 was considered statistically significant.

## 3 RESULTS

### 3.1 CC‐5013 ameliorated pathological damage in ALF and AKI mice

**Figure 1:**
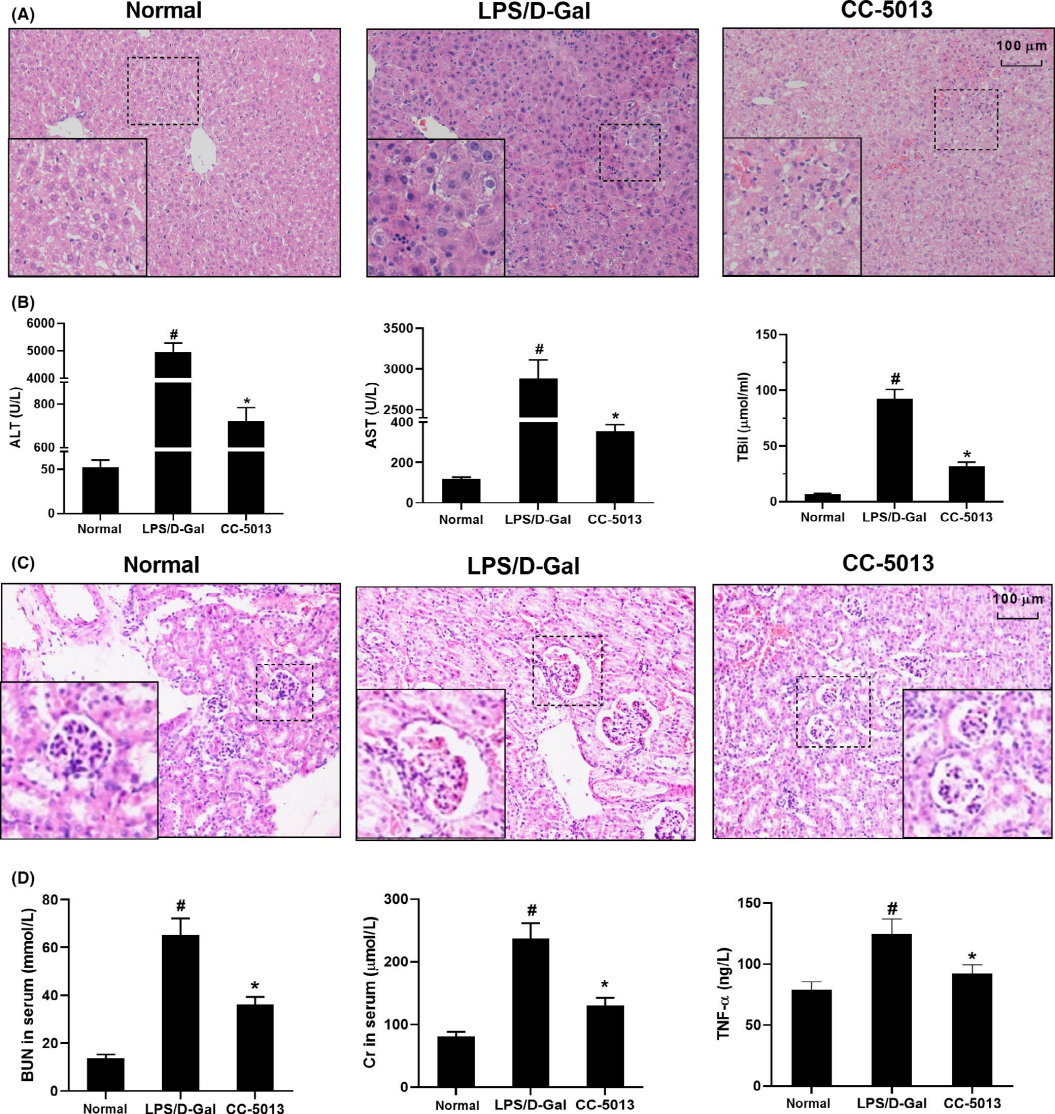
CC‐5013 ameliorated tissue damage in the ALF and AKI mouse model. A, Pathological changes in liver tissues were examined by H&E staining. B, The serum ALT, AST and TBil levels were determined in different groups. C, Pathological changes in kidney tissues were examined by H&E staining. D, Serum BUN, Cr and TNF‐α levels were detected in different groups. Data are shown as mean ± SD. n = 10. #*P* < .05, compared with the normal group. *P < .05, compared with the LPS/D‐Gal group

As shown in Figure 1A, in the normal group, the structure of liver lobules was clear and the hepatocytes were arranged neatly. There was no inflammatory cell infiltration around the hepatocytes. In the model group, the hepatic lobular structure was blurred; the hepatocytes were massively necrotic and were surrounded by inflammatory cell infiltration. However, the structure of hepatic lobules in the CC‐5013 group was clearer, and necrosis of hepatocytes was significantly reduced compared to that in the model group. Moreover, inflammatory cell infiltration was also significantly reduced. As shown in Figure 1B, the serum ALT, AST and TBil levels in the model group were higher compared to those in the normal group (*P* < .05). After administration of CC‐5013, the serum ALT, AST and TBil levels decreased (*P* < .05). As shown in Figure 1C, histology of the kidney in the normal group presented a clear structure of renal tubules and glomeruli. By contrast, the kidney in the model group exhibited swollen tubular epithelial cells with indistinct brush borders and vacuoles in tubular cells. CC‐5013 ameliorated swelling and vacuolar degeneration in the tubular cells. As shown in Figure 1D, the serum BUN, Cr and TNF‐α levels in the model group were higher than those in the normal group (*P* < .05). However, after administration of CC‐5013, the serum BUN, Cr, and TNF‐α levels were decreased compared to those in the model group (*P* < .05).

### 3.2 CC‐5013 inhibited HMGB1 production and pyroptosis in ALF and AKI mice

**Figure 2:**
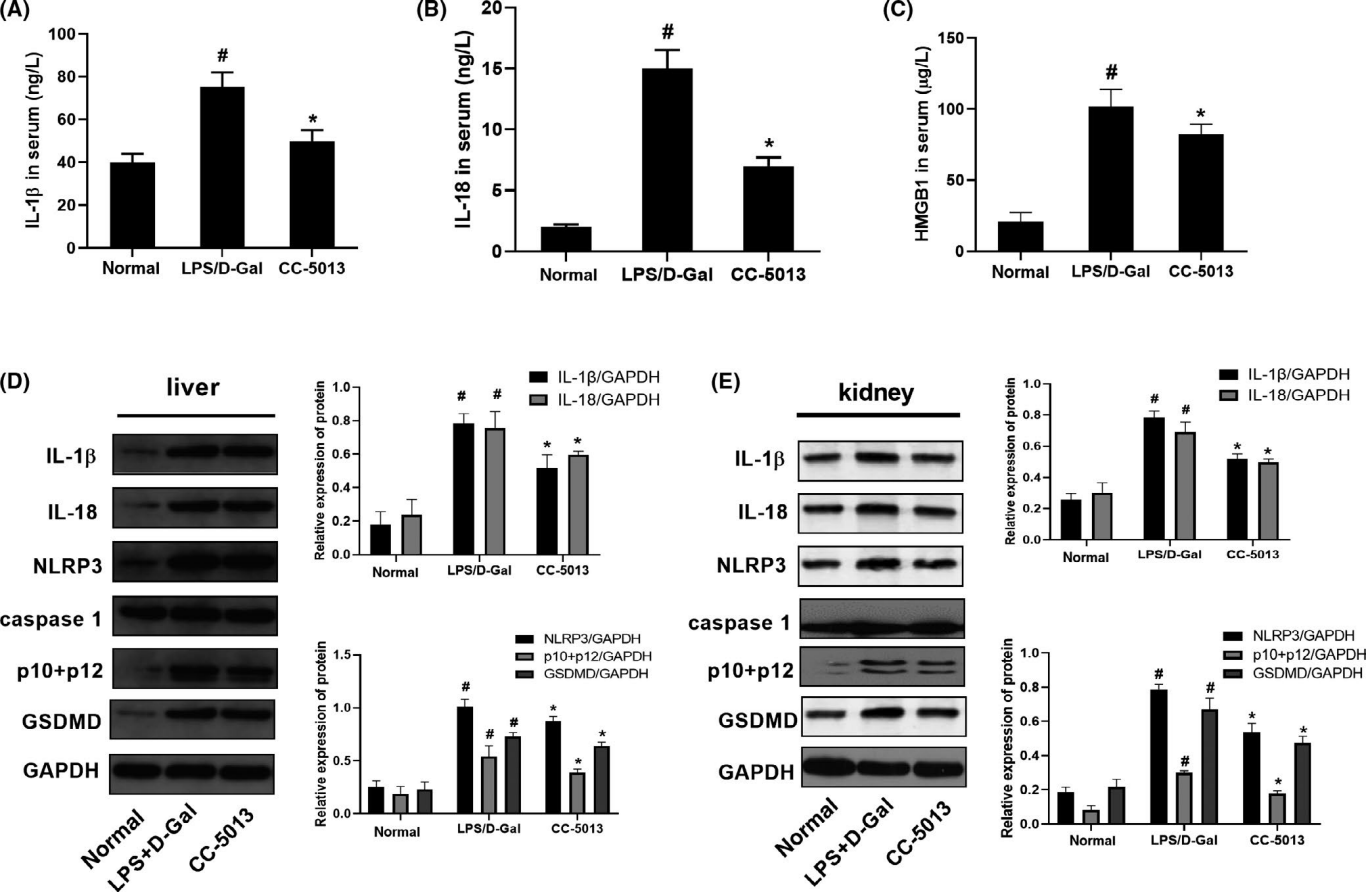
CC‐5013 inhibited HMGB1 and pyroptosis in the ALF and AKI mouse model. A‐C, Serum levels of HMGB1, IL‐1β, IL‐18, ALT, AST and TBil were determined in different groups. D‐E, Protein levels of IL‐1β, IL‐18, NLRP3, p10 + p12 and GSDMD in liver and kidney tissues were detected by Western blotting in different groups. Data are shown as mean ± SD. n = 10. # *P* < .05, compared with the normal group. *P < .05, compared with the LPS/D‐Gal group

The serum concentrations of HMGB1, IL‐1β, IL‐18 in the model (ALF and AKI) group were higher compared to those in the normal group (*P* < .05). After administration of CC‐5013, the concentrations of HMGB1, IL‐1β and IL‐18 were decreased **(** *P* < .05; Figure 2A‐C). Moreover, the protein levels of IL‐1β, IL‐18, NLRP3, p10 + p12 and GSDMD in the model (AKI and ALF) group were higher compared to those in the normal group (*P* < .05; Figure 2D,E). After administration of CC‐5013, the protein levels of IL‐1β, IL‐18, NLRP3, p10 + p12 and GSDMD in the treated model group were decreased compared to those in the untreated model group (*P* < .05).

### 3.3 CC‐5013 inhibited activation of M1 macrophages in liver and kidney tissues

M1 macrophages play a pro‐inflammatory role in ALF and AKI. In the process of pyroptosis, inflammatory factors are released along with cell damage; M1 macrophages are the main source of pro**‐**inflammatory factors.

**Figure 3:**
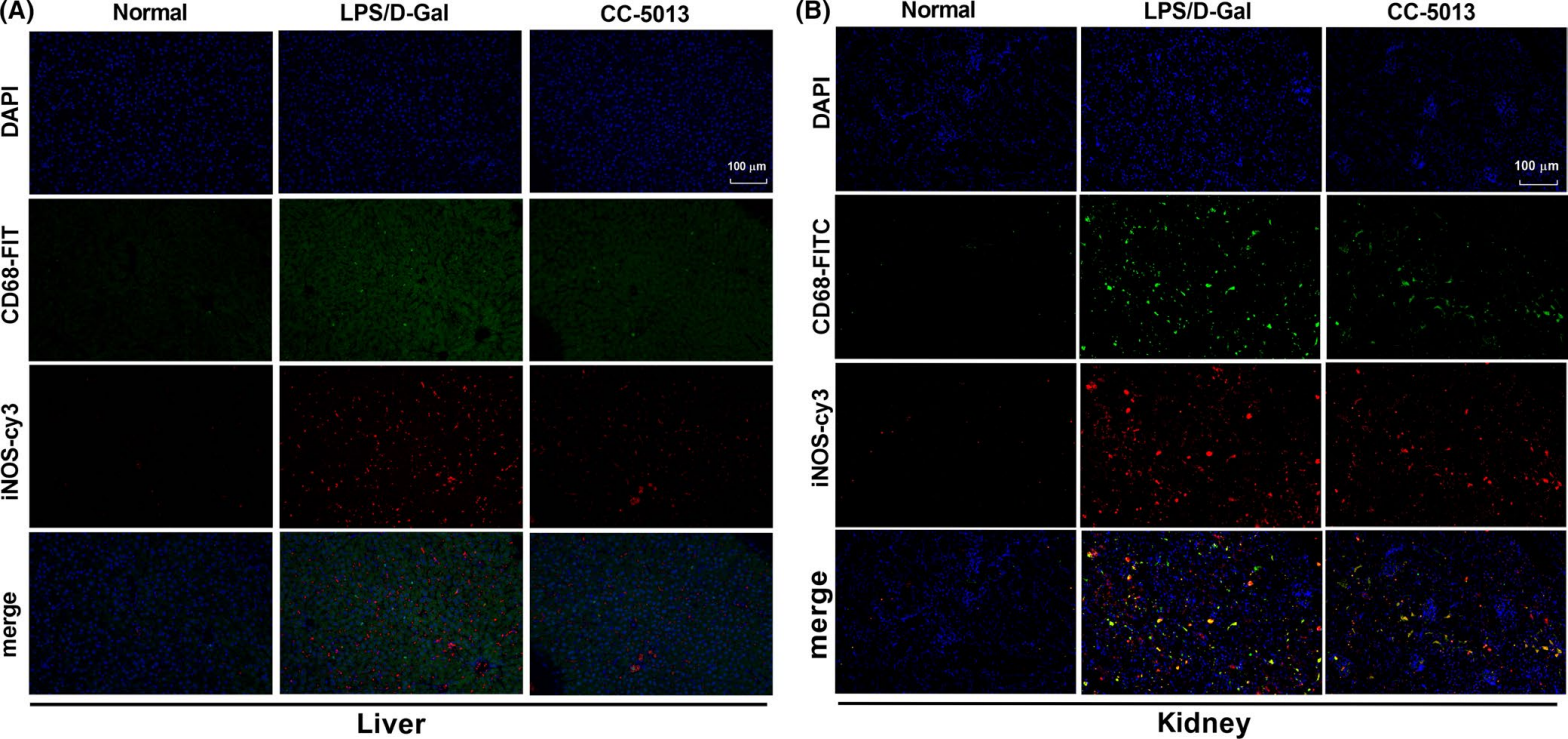
CC‐5013 inhibited the activation of M1 macrophages in liver and kidney tissues. A, B, Expression levels of marker proteins CD68 and iNOS of M1 macrophages in liver and kidney sections were detected by immunofluorescence

The two biomarkers, CD68 and iNOS, are widely used to study the effects of M1 macrophages in kidney and liver.^27^, ^28^ As shown in Figure 3, the expression levels of the marker proteins CD68 and iNOS in M1 macrophages of the liver and kidney tissues of the model group were higher compared to those in the normal group. However, after administration of CC‐5013, the protein levels of CD68 and iNOS decreased compared to those in the model group.

### 3.4 PMA stimulated the differentiation of THP‐1 cells into macrophages and LPS induced the differentiation of M1 macrophages

**Figure 4:**
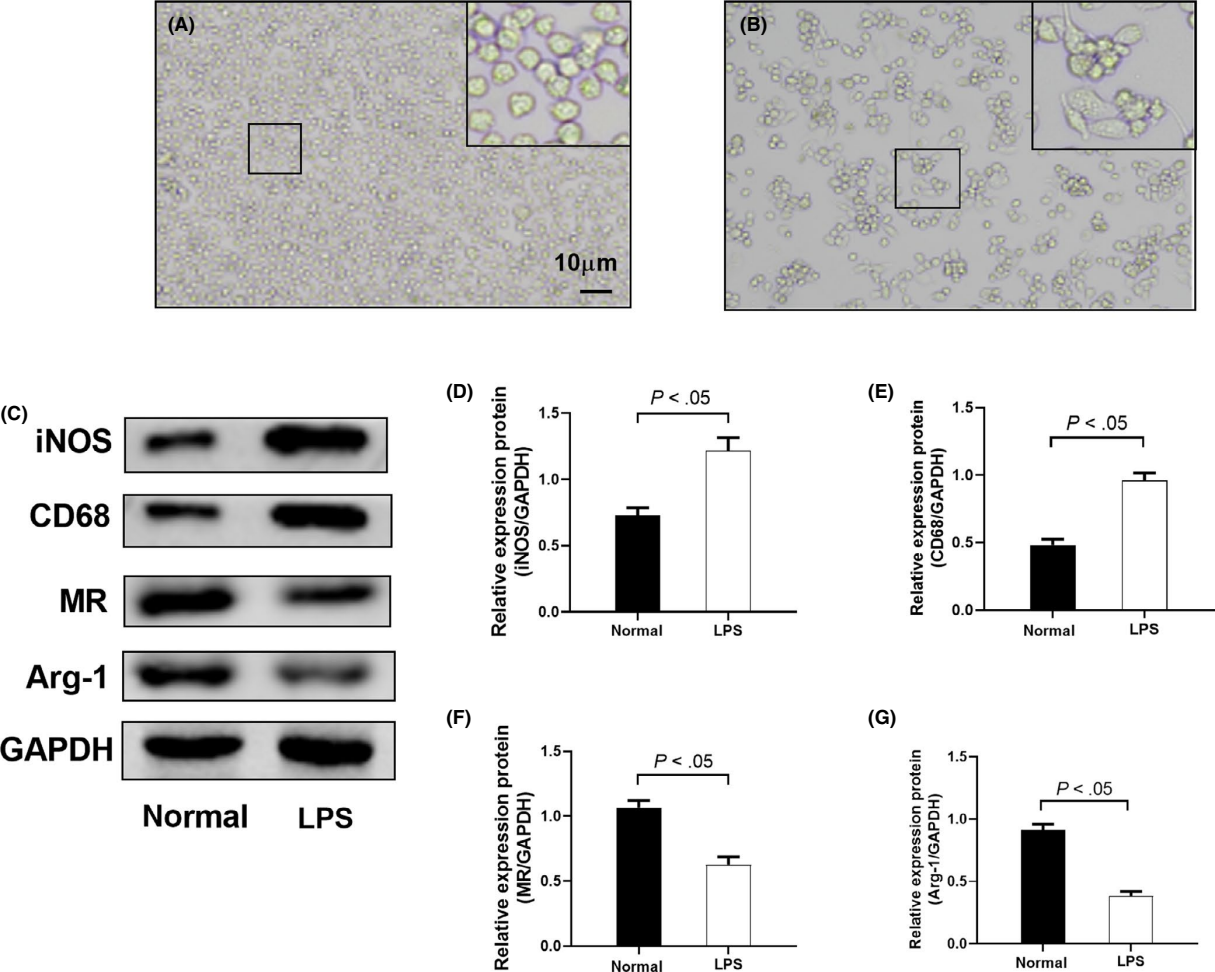
PMA stimulated differentiation of THP‐1 into macrophages, and LPS induced further differentiation of M1 macrophages. A, Morphology of THP‐1 cells. B, Morphology of PMA‐stimulated THP‐1 cells. C‐G, Protein levels of iNOS, CD68, MR and Arg‐1 were detected by Western blotting. Data are shown as mean ± SD. n = 3

A large number of M1 macrophages are activated during liver and kidney injury; hence, a common monocyte–macrophage THP‐1 cell line was activated by PMA and LPS to be transformed into M1 type macrophages. As shown in Figure 4A,B, THP‐1 cells became rounded and suspended. Compared to those in the normal group, cells in the PMA‐stimulated group adhered to the wall of the flask, significantly fewer round cells were observed, and some cells extended pseudopods and exhibited dendritic and radial shapes. As shown in Figure 4C‐G, the expression of the M1 macrophage markers iNOS and TNF‐α proteins in the LPS‐stimulated group was significantly increased compared to that in the normal group (*P* < .05), while the expression of M2 macrophage markers MR and Arg‐1 decreased (*P* < .05).

### 3.5 TNF‐α SNPs affected HMGB1 and pyroptosis pathways in M1 macrophages

**Figure 5:**
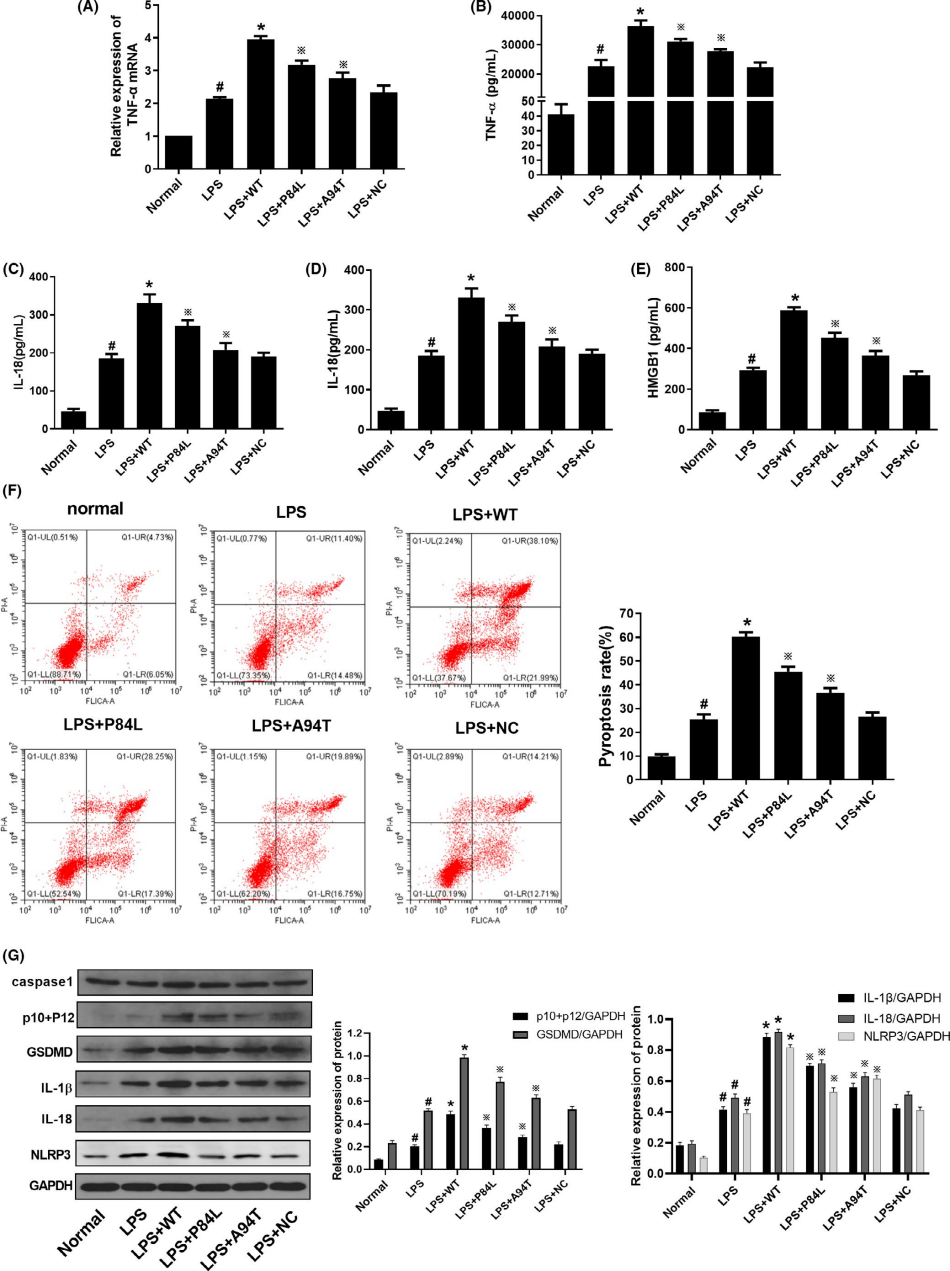
TNF‐α SNPs decreased the expression of TNF‐α, IL‐1β and IL‐18 in M1 macrophages. A, mRNA levels of TNF‐α were detected by RT‐PCR. B‐E, Concentrations of TNF‐α, IL‐1β, IL‐18 and HMGB1 in cell supernatants were detected by ELISA. F, Pyroptosis rates were determined by flow cytometry. G, Protein levels of pro‐caspase‐1 + p10 + p12, GADMD, IL‐1β, IL‐18 and NLRP3 were detected by Western blotting. Data are shown as mean ± SD. n = 3. #*P* < .05, compared with the normal group. *P < .05, compared with the LPS group. ※*P* < .05, compared with the LPS + WT group

As shown in Figure 5A‐F, the concentrations of TNF‐α, HMGB1, IL‐1β and IL‐18 in cell supernatants; mRNA expression of TNF‐α; and pyroptosis rates were higher in the LPS group compared to those in the normal group (*P* < .05). Similarly, the concentrations of TNF‐α, HMGB1, IL‐1β and IL‐18; mRNA expression of TNF‐α; and pyroptosis rates were higher in the LPS + WT group than in the LPS group (*P* < .05). By contrast, compared with those in the WT group, the concentrations of TNF‐α, HMGB1, IL‐1β and IL‐18; mRNA expression of TNF‐α; and pyroptosis rates were lower in the LPS + A94T and LPS + P84L groups (*P* < .05). However, there was no difference observed in the activation of HMGB1 and pyroptosis pathways between the LPS and LPS + NC groups.

### 3.6 TNF‐α SNPs decreased protein expression associated with pyroptosis in M1 macrophages

The protein levels of p10 + p12, GADMD, IL‐1β, IL‐18 and NLRP3 in the LPS group were increased compared to those in the normal group (*P* < .05); moreover, these levels were increased in the LPS + WT group compared with the LPS group (*P* < .05). By contrast, the protein levels of p10 + p12, GADMD, IL‐1β, IL‐18 and NLRP3 were decreased in the LPS + A94T and LPS + P84L groups compared to those in the WT group (*P* < .05). However, there was no difference in the molecules related to pyroptosis between the LPS and LPS + NC groups (Figure 5G).

### 3.7 Glycyrrhizin inhibited pyroptosis in the TNF‐α‐induced M1 macrophages

**Figure 6:**
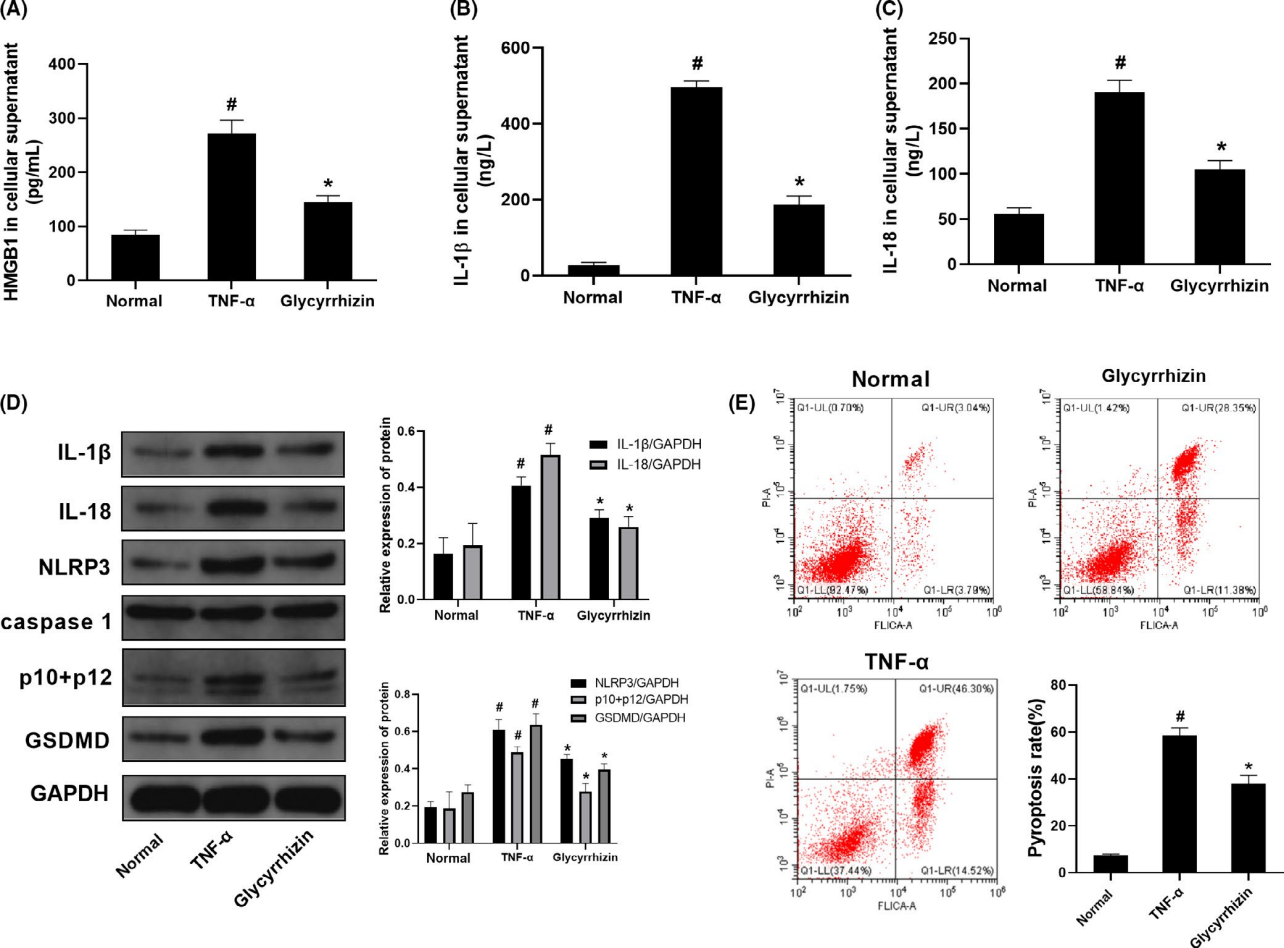
Glycyrrhizin inhibited pyroptosis in TNF‐α‐induced M1 macrophages. A‐C, Concentrations of HMGB1, IL‐1β and IL‐18 in cell supernatants were detected by ELISA. D, Protein levels of pro‐caspase‐1 + p10 + p12, GADMD, IL‐1β, IL‐18 and NLRP3 were detected by Western blotting. E, Pyroptosis rates were detected by flow cytometry. Data are shown as mean ± SD. n = 3. #*P* < .05, compared with the normal group. *P < .05, compared with the TNF‐α group

The concentrations of HMGB1, IL‐1β and IL‐18 in the cell supernatants of the TNF‐α group were higher than those of the normal group (*P* < .05). After administration of glycyrrhizin, the concentrations of HMGB1, IL‐1β and IL‐18 were observed to be lower compared to those in the TNF‐α group (*P* < .05; Figure 6A‐C). The protein levels of IL‐1β, IL‐18, NLRP3, p10 + p12 and GSDMD were higher in the TNF‐α group compared to those in the normal group (*P* < .05). When compared with those in the TNF‐α group, the protein levels of IL‐1β, IL‐18, NLRP3, p10 + p12 and GSDMD in the glycyrrhizin group were decreased (*P* < .05; Figure 6D). Regarding the pyroptosis rate, the pyroptosis level in the TNF‐α group was higher compared to that in the normal group (*P* < .05). By contrast, the pyroptosis level in the glycyrrhizin group was lower than that in the TNF‐α group (*P* < .05; Figure 6E).

## 4 DISCUSSION

AKI is one of the serious complications in patients with liver failure. Previous studies have reported that the incidence of AKI in ALF is as high as 38%‐70%.^29^, ^30^ Hepatorenal syndrome (HRS) is the terminal form of AKI. It has a high mortality rate and can lead to a prolonged hospital stay.^31^ Treatments, such as the use of vasoconstrictors, albumin, transjugular intrahepatic portosystemic shunt, as well as liver and liver–kidney transplantations, are the available strategies to prevent this serious complication in recent times.^32^ The development of better biomarkers for kidney damage, early diagnosis and timely intervention of AKI in ALF is extremely important to reduce mortality and improve the prognosis of liver failure.

The pathogenesis of AKI in ALF is still unknown. Currently, systemic haemodynamics, systemic inflammation, immune dysfunction and bacterial infections are recognized as risk factors.^33^ Increasing evidences suggest that inflammation leads to the release of pro‐inflammatory cytokines and chemokines, which play an important role in the development of AKI in ALF.^34^ Patients with ALF have low immune function, decreased intestinal mucosal barrier function, intestinal bacterial overgrowth, and consequently are prone to various infections.^7^ On the one hand, inflammatory mediators directly bind to the pattern recognition receptors, such as Toll‐like receptor (TRL) 2 and TRL 4 on hepatocytes and renal tubular cells, resulting in cell damage. On the other hand, these inflammatory mediators can also activate immune cells (such as macrophages) in the liver and kidneys, which could induce oxidative stress, microcirculation and mitochondrial metabolic disorders. These phenomena eventually cause apoptosis or necrosis of hepatocytes and renal tubular cells.^6^, ^35^, ^36^

LPS has a low specificity for liver injury; hence, it is often combined with D‐Gal to establish an animal model of acute inflammatory liver injury.^37^ Studies have shown that D‐Gal can deplete uridine triphosphate (UTP) via the galactose pathway to inhibit protein synthesis, subsequently producing reactive oxygen species (ROS) to induce liver damage. Therefore, D‐Gal can be used as a sensitizer in LPS‐induced liver injury in vivo.^38^ Currently, injection of LPS combined with D‐Gal simulates an acute inflammatory liver injury model, which is widely accepted and used to explore and develop new liver‐protective agents for inflammatory liver injury.^39^ In this study, acute kidney injury also occurred in the animal models using liver failure‐specific causative agents.

In the process of inflammatory cell‐activation during ALF and AKI, innate immune cells, such as macrophages, which are widely present in the liver, kidneys, and other organs play an important role. Under normal circumstances, macrophages exist in a resting state in low numbers and have a low metabolic rate and long survival time.^40^ M1 macrophages are polarized by the stimulation of pro‐inflammatory factors, such as infections, endotoxins and hypoxia. Under these conditions, the cell volume is enlarged, and cell metabolism is activated with the production of large amounts of TNF‐α, IL‐6 and iNOS.^41^ M2 macrophage polarization occurs under the stimulation of IL‐4 and IL‐10. In this case, the mannose receptor (MR) is increased, and a large number of anti‐inflammatory factors, such as IL‐10, Arg‐1 and IL‐13, are secreted by the polarized M2 macrophages. Cytokines can exert effects of anti‐inflammation and tissue repair.^42^ Therefore, M1 macrophage polarization and their mediated inflammatory responses are the main factors that cause cell damage.^43^ More importantly, TNF‐α released by M1 macrophages could further stimulate inflammatory cells and aggravate the inflammatory response, leading to a vicious cycle.^10^ Therefore, controlling the release of TNF‐α by M1 macrophages is the key to control cell damage.

Pyroptosis is a programmed cell death pattern that is different from necrosis and apoptosis.^44^ NOD‐like receptors (NLRs), such as NLRP3, play an important role in the recognition of danger signals in the host cytoplasm during pyrotosis. One part of the NLR recognizes foreign pathogens and releases inflammatory cytokines, such as TNF‐α, IFN‐α and IL‐12. The other part of NLR mediates the activation of caspase 1, which triggers caspase 1‐dependent pyroptosis and releases inflammatory cytokines IL‐18 and Il‐1β.^45^ At the same time, a component of the inflammasome, GSDMD, is sheared and participates in the formation of cell membrane pores; the inflammatory mediators IL‐18 and Il‐1β can be released into the extracellular space through these pores.^46^ As a result, pyroptosis can lead to an excessive inflammatory response, accumulation of inflammatory mediators and infiltration by inflammatory cells, finally leading to fever, low blood pressure and other clinical manifestations.^47^, ^48^

As shown in the present study, CC‐5013 ameliorated pathological damages to the liver and kidneys in the ALF and AKI mouse model. Liver and renal functions were also significantly improved. More importantly, CC‐5013 inhibited serum HMGB1 levels. The levels of molecules related to pyroptosis, such as IL‐1β, IL‐18, NLRP3, p10 + p12 and GSDMD in the liver and kidney tissues of the ALF and AKI mouse model, were also decreased. To further investigate whether M1 type macrophages participate in the process of pyroptosis in liver and kidney tissues, we also examined the marker proteins CD68 and iNOS of M1 macrophages in the liver and kidneys, respectively. After administration of CC‐5013, the CD68 and iNOS levels in the model group were decreased. Taken together, these results suggest that TNF‐α can act as an upstream regulatory molecule that causes pyroptosis in liver and kidney tissues, activates M1 macrophages, and releases HMGB1.

Inflammation is the main cause of liver failure and acute kidney injury. M1 macrophages play a pro‐inflammatory role in ALF and AKI. In the process of pyroptosis, inflammatory factors are released along with cell damage; M1 macrophages are the main source of pro‐inflammatory factors. The present study was conducted from the perspective of M1 macrophage activation to explore the effect of the TNF‐α/HMGB1 pathway on pyroptosis in liver failure and acute kidney injury.

Due to unavailability of specific Kupffer cell lines or kidney macrophages, after confirming that a large number of M1 macrophages are activated in liver and kidney injury, a common monocyte–macrophage THP‐1 cell line was further activated by PMA and LPS to be transformed into M1 type macrophages (Figure 3). It is universally acknowledged that the PMA‐stimulated human monocyte cell line, THP‐1, can differentiate into macrophages. Hence, it is extensively used in vitro as a cell model to study macrophages.^49^, ^50^ In an in vitro study, PMA was first used to stimulate differentiation of THP‐1 cells into macrophages. LPS was then used to induce differentiation of M1 macrophages. Compared with that of normal macrophages, the expression of M1 macrophage markers, iNOS and TNF‐α, in the LPS‐stimulated group was increased. However, the expression of M2 macrophage markers, MR and Arg‐1, was decreased. These results demonstrated that the M1 macrophage model was successfully generated in this study. Later, our results demonstrated that the TNF‐α inhibitor, CC‐5013, decreased the expression of TNF‐α, IL‐1β and IL‐18 in M1 macrophages. Compared with those in the LPS group, the concentrations of TNF‐α, IL‐1β, IL‐18 and HMGB1, and mRNA levels of TNF‐α were increased in the LPS + WT group. Compared with those in the WT group, the concentrations of TNF‐α, IL‐1β, IL‐18 and HMGB1, and mRNA levels of TNF‐α were decreased in the LPS + A94T and LPS + P84L groups. TNF‐α SNPs decreased the pyroptosis rate of M1 macrophages. The pyroptosis rate was lower in the LPS + A94T and LPS + P84L groups than in the WT group. We also observed that the TNF‐α SNPs decreased the expression of molecules that cause pyroptosis in M1 macrophages. Compared with those in the LPS group, the protein levels of p10 + p12, GADMD, IL‐1β, IL‐18 and NLRP3 were higher in the LPS + WT group. By contrast, the protein levels of p10 + p12, GADMD, IL‐1β, IL‐18 and NLRP3 were lower in the LPS + A94T and LPS + P84L groups compared to those in the WT group. However, there was no difference between the LPS group and the LPS + NC group regarding molecules related to pyroptosis. The reason for the two variants A94T and P84L affecting the pyroptosis molecules may due to the fact that TNF‐α SNPs destabilize amino acid interactions and hydrogen bond networks, which in turn affect the expression of TNF‐α.^51^, ^52^ These results indicated that TNF‐α could induce pyroptosis in M1 macrophages and the release of HMGB1.

To further investigate whether HMGB1 could act as an intermediary molecule in tandem with TNF‐α and pyroptosis, we initially used TNF‐α to induce pyroptosis in M1 macrophages and later intervened the process of pyroptosis with the HMGB1 inhibitor, glycyrrhizin. After administration of glycyrrhizin, the concentrations of HMGB1, IL‐1β and IL‐18 in cell supernatants; the protein levels of IL‐1β, IL‐18, NLRP3, p10 + p12 and GSDMD; and pyroptosis rate were decreased.

In conclusion, systemic inflammatory responses play a pivotal role in the pathogenesis of AKI in ALF patients. Inhibition and mutation of TNF‐α could suppress the effects of HMGB1, thereby inhibiting the process of pyroptosis. The TNF‐α/HMGB1 inflammation signalling pathway plays an important role in pyroptosis during liver failure and AKI. Moreover, the TNF‐α SNPs may also inhibit the M1 macrophage pyroptosis. Hence, in addition to the TNF‐α inhibitors or neutralizing antibodies that are currently used for treatment, a combination of HMGB1 inhibitors or TNF‐α SNPs can also be considered for the treatment of liver failure and acute kidney injury. Taken together, these data provide a scientific basis to better understand the pathogenesis of ALF and AKI and to design novel treatment strategies for these debilitating conditions.

## CONFLICTS OF INTEREST

The authors declare that they have no conflicts of interests.

## AUTHOR CONTRIBUTIONS

Zuojiong Gong takes responsibility for the integrity of the work as a whole, from inception to published article. Yao Wang, Hong Zhang and Zuojiong Gong conceived and designed the experiments. Yao Wang, Haiyue Zhang, Fangzhou Jiao, Chunxia Shi and Qian Chen performed the experiments. Yao Wang, Haiyue Zhang and Luwen Wang analysed the data. Qian Chen, Chunxia Shi, Maohua Pei and Jian Lv contributed reagents/materials/analysis tools. Yao Wang wrote the paper. Zuojiong Gong edited the article. All authors approved the final version of the manuscript.

## Data Availability

The data used to support the findings of this study are available from the corresponding author upon request.
